# Pharmacogenomics in the prediction of adverse effects of cardiovascular drugs: the PGx-CardioDrug project

**DOI:** 10.3325/cmj.2025.66.446

**Published:** 2025-12

**Authors:** Tamara Božina, Lana Ganoci, Livija Šimičević, Majda Vrkić Kirhmajer, Iva Mucalo, Jure Samardžić, Jozefina Palić, Ana Marija Slišković, Vladimir Trkulja

**Affiliations:** 1 Department of Medical Chemistry, Biochemistry and Clinical Chemistry, Zagreb University School of Medicine, Zagreb, Croatia; 2 Division for Pharmacogenomics and Therapy Individualization, Department of Laboratory Diagnostics, University Hospital Center Zagreb, Zagreb, Croatia; 3 Department of Basic and Clinical Pharmacology, Zagreb University School of Medicine, Zagreb, Croatia; 4 Department of Cardiovascular Diseases, University Hospital Center Zagreb, Zagreb, Croatia; 5 Center for Applied Pharmacy, University of Zagreb Faculty of Pharmacy and Biochemistry, Zagreb, Croatia; 6 Department of Cardiovascular Diseases, Zagreb University School of Medicine, Zagreb, Croatia; 7 Department of Laboratory Diagnostics, University Hospital Center Zagreb, Zagreb, Croatia; 8 Department of Internal Medicine, Zagreb University School of Medicine, Zagreb, Croatia

## Abstract

Although the role of pharmacogenomics (PGx) in personalized pharmacotherapy has been well established, its implementation in clinical practice lags behind. In this article, we present an overview of important achievements in the field of PGx of cardiovascular drugs (CVDs), and identify gaps in the existing research. We also present an outline of the PGx-CardioDrug project (ClinicalTrials.gov: NCT05307718) focused on PGx of three major classes of CVDs: P2Y12 antiplatelets, direct oral anticoagulants (DOACs), and statins. The project intends to evaluate pharmacogenes, concomitant treatments, and their combinations as determinants of adverse drug reactions (ADRs). It is based on a pool of around 1200 consecutive adults who were accrued on the case-control principle defined with respect to the CVDs and ADRs of interest (bleeding related to antiplatelets and DOACs, skeletal muscle or liver toxicity related to statins, inefficiency). Possible perpetrator or victim roles of concomitantly used drugs are assessed using the Lexicomp^®^ Clinical Decision Support System. The assembled data provide a basis for a series of case-control type analyses. Considering the mode of data generation and the nature of the exposures of interest (ie, present before the occurrence of the outcome), potentially observed outcome-exposure associations are likely to be causal, provided that confounding is reasonably controlled. The project enables the development of methods and procedures that better reflect the real-life situation of patients with comorbidities and polytherapy, and might better predict the interactions of multiple drugs and genes that affect the frequency and severity of CVD ADRs.

Pharmacogenomics (PGx) has enhanced our ability to relate the exposure to and effects of a drug to particular gene variants. Consequences of genetic variations range from clinical inefficacy to serious adverse drug reactions (ADRs) (1). Genes can modulate drug effects (beneficial or harmful) by affecting pharmacokinetics (PK) (ie, the activity of systems responsible for drug absorption, distribution, metabolism, and elimination [ADME genes]) or pharmacodynamics (PD) (genetically determined alterations in target molecules). Up to 40% of unexpected drug reactions are driven by the genetic background – PGx clearly has an important role in the advancement of personalized medicine by optimizing drug selection and dosage (2).

The establishment of the Clinical Pharmacogenetics Implementation Consortium (www.cpicpgx.org) in 2009 by the National Institutes of Health and the Pharmacogenomics Knowledge Base (PharmGKB, http://www.pharmgkb.org) created the prerequisites for the publication of freely available, peer-reviewed, evidence-based, and updated guidelines for the implementation of PGx in clinical practice (3).

Recent studies have demonstrated that genotype-guided treatment can effectively reduce the incidence of adverse drug events by up to 30% (4). Cardiovascular diseases are a leading cause of morbidity and mortality worldwide. Genetic variations have been identified that impact PK, efficacy, and adverse effects of major cardiovascular drugs (CVDs), such as renin-angiotensin-aldosterone system inhibitors, calcium channel blockers, antiplatelets, diuretics, statins, beta-blockers, and anticoagulants (5,6). Commonly, several CVDs are used concomitantly, thus increasing the risk of practically relevant drug-drug interactions, and – as it is becoming ever more obvious – practically relevant complex interactions between several drugs and gene variants (ie, drug-drug-gene interactions) (7,8).

PGx information may be important with respect to almost 70% of the CVDs (9,10). Thus far, guidelines and recommendations for drug dosing have been issued for coumarin anticoagulants phenprocoumon (11,12) and warfarin (13), propafenone (11,12), clopidogrel (14), and some statins (15). While PGx holds potential for improving the management of cardiovascular diseases by enabling personalized approaches tailored to individual genetic profiles, challenges such as clinical implementation, cost-effectiveness, and ethical considerations need to be addressed before pharmacogenomic testing can be completely incorporated into standard clinical practice. Continued research and clinical analysis are necessary to improve therapeutic outcomes and reduce the burden of cardiovascular diseases worldwide (16-18); hence, a need for population-based research has been emphasized having in mind ethnic and racial differences in the frequency of potentially relevant genetic variants (19,20). We here present an outline of the PGx-CardioDrug project (ClinicalTrials.gov: NCT05307718) focused on the implementation of PGx in the improvement of safe use of three major classes of CVDs.

## PGX-CardioDrug project

The project is focused on three classes of CVDs that are commonly used concomitantly, are inherently prone to mutual drug-drug interactions (DDIs), and are also susceptible to variability in exposure and activity driven by variants in the ADME genes – antiplatelets of the adenosine P2Y12 receptor antagonist class (specifically clopidogrel, ticagrelor, and prasugrel), direct oral anticoagulants (DOACs) (thrombin inhibitor dabigatran; factor Xa inhibitors rivaroxaban, apixaban, and edoxaban), and statins (7,21,22). The project intends to evaluate pharmacogenes, concomitant treatments, and their combinations as determinants of adverse effects.

## Current knowledge and challenges

P2Y12 antiplatelets are susceptible to both pharmacodynamic (eg, other antiplatelets, anticoagulants) and pharmacokinetic DDIs, with a propensity of being affected by the ADME genes. Clopidogrel is an inactive prodrug, whose gastrointestinal absorption is limited by the efflux transporter P-glycoprotein (P-gp, ABCB1, *ABCB1* gene) (23) and requires activation by several isoenzymes of the cytochrome P-450 superfamily – CYP2C19 (major; strongly inhibited by some of the common concomitant treatments, such as omeprazole and esomeprazole) (24,25), 1A2, 2B6, 2C9, and 3A4. Clopidogrel is metabolized (as the primary route of elimination) by carboxylesterase 1 (CES1) (26). One of the challenges of PGx research is investigation into the role of gain-of-function alleles as potential predictors of bleeding with clopidogrel use. Currently, guidelines for clopidogrel refer only to the role of the *CYP2C19* loss-of-function alleles (*2*3) (14), while the significance of the allele of enhanced function (*^17^) is not entirely clear. Increased bleeding risk has been suggested in *17 carriers (rapid [RM] and ultrarapid metabolizers [UM]) (27-30), but this is still dubious (31,32). Recently, a novel haplotype *CYP2C-TG* in the *CYP2C* gene cluster was identified and reported to be associated with the ultrarapid metabolism of CYP2C19 substrates escitalopram and sertraline (33,34). However, its role in enzyme activity *in vitro*, and the metabolism of substrate drugs has not been unequivocally confirmed (35-37). Data on the association between *CYP2C-TG* haplotype and variability in clopidogrel efficacy and bleeding risk are lacking. Preliminary data by the collaborators of the PGx-CardioDrug project indicated more ischemic events and fewer bleeding events in poor and intermediate metabolizers than in normal and combined RM/UM patients (38). Ticagrelor is a substrate to and a weak inhibitor of ABCB1. It is eliminated predominantly by CYP3A4. Combined *in vitro* and in vivo data suggest that ticagrelor may be both a weak inhibitor and activator of CYP3A (39). The CYP3A4*22 variant may increase bleeding risk in ticagrelor users (40), but this has not been confirmed (41). More recent data report on interactions between ticagrelor and statins at the level of CYP3A4 and drug transporters, which increases the toxicity of statins (42-45). Ticagrelor does not seem to be relevantly affected by genetic variants in *CYP2C19* or *ABCB1* genes, *SLCO1B1* (codes for the organic anion transporting polypeptide B1, OAT1B1) or *UGT2B7* genes (codes for the UDP-glucoronosyltransferase isoenzyme 2B7) (46).

DOACs are characterized by marked interindividual variations in exposure. The findings to date indicate drug interactions, as well as PGx, as likely causes, but knowledge is still very limited (47,48). Rivaroxaban is a substrate of CYP3A4, CYP2C8, and CYP2J2 enzymes, ABCB1, and breast cancer resistance protein (ABCG2, *ABCG2* gene) efflux transporters. Thus far, PGx studies (including those by the project collaborators) have suggested an association between CYP3A enzyme activity and rivaroxaban concentration, with a possible role of the polymorphic efflux transporters ABCB1 and ABCG2 (47,49-51). Apixaban is mainly metabolized by CYP3A4, and is a substrate of ABCB1 and ABCG2. Pharmacogenes that challenge further research are *CYP3A4*, *ABCB1,* and *ABCG2* (52). Dabigatran etexilate is a prodrug converted to its active form by CES1, while its absorption is affected by ABCB1. *ABCB1* (rs1045642, rs4148738) and *CES1* variants (rs2244613, rs8192935) contribute to interindividual differences in plasma dabigatran concentrations, but their significance for clinical outcomes has not yet been confirmed (53-55). Edoxaban is metabolized by CES1, CYP3A4, and glucuronidation, and is a substrate of ABCB1 (56). One of its metabolites is a substrate of the OATP1B1 transporter (*SLCO1B1* gene). The findings to date suggest a possible role of *CYP3A4*, *CES1*, *SLCO1B1*, and *ABCB1* gene variants in exposure to edoxaban (57).

Statins are generally well tolerated, but their possible interactions with other drugs may increase the risk of statin myopathy and hepatotoxicity. These interactions may be caused by polypharmacy and pharmacogenetic variability (58). Statins are metabolized by CYPs (CYP3A4, CYP2C9, and CYP2C19) and are substrates of membrane transporters OATP1B1, ABCG2, ABCB1, and ABCC2 (multidrug resistance-associated protein 2, ABCC2 transporter, *ABCC2* gene). The current pharmacogenetic guidelines for statin dosing refer to *SLCO1B1, ABCG2* and *CYP2C9* genotypes (15).

## General design

**Figure 1:**
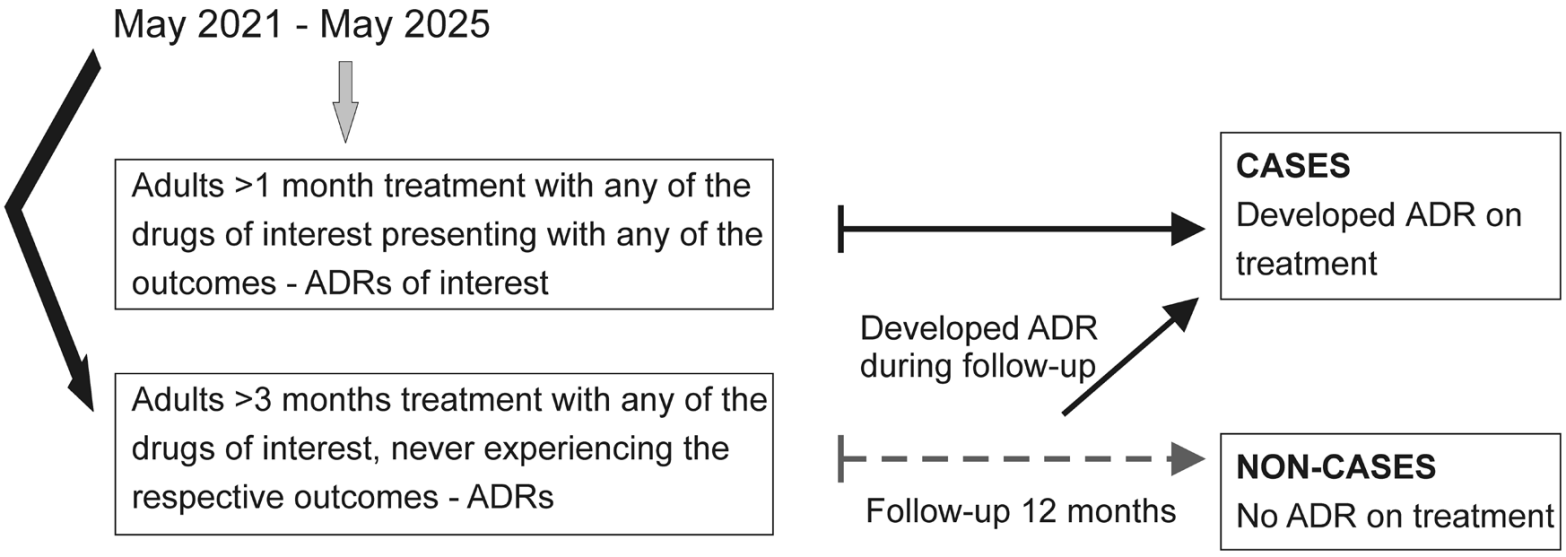
The inclusion criteria for cases and controls. ADR – adverse drug reactions.

The project is based on a pool of around 1200 consecutive adult patients managed over a 3.5-year period across 3 participating hospitals in Croatia, who were accrued on a case-control principle defined with respect to the CVDs of interest (individual P2Y12 antiplatelets, DOACs, statins) and ADRs of interest (bleeding related to antiplatelets and DOACs, skeletal muscle or/and liver toxicity related to statins). The general case-control principle is as follows: i) patients treated with any (one or more) of the CVDs of interest for at least 1 month and presenting with respective ADRs are considered “cases;” ii) their contemporary peers, ie, prevalent users of the same drug of interest for at least 3 months without experiencing ADRs are included and followed up over the subsequent 12-month treatment period (Figure 1). Those who develop the respective ADRs are included in the “pool of cases,” while others are considered non-cases. Exposures of interest are pharmacogenes (as genotypes or genotype-predicted phenotypes) and concomitant medications (eg, enzyme/transporter inhibitors or inducers, or pharmacodynamic perpetrator drugs) or their combinations. Since the case/non-case classification is drug-ADR specific, a patient treated with two or more CVDs of interest could be a “case” in a case-control analysis of association between the exposure of interest and one type of ADRs, and a control in a case-control analysis relating exposure of interest to another type of ADR. For cases, PGx (blood sample for genotyping) and pharmacological exposures (concomitant drugs: treatment over at least 5 days preceding the ADR occurrence) are determined at the time of ADR occurrence. For non-cases, PGx and pharmacological exposures are determined at the start of the 12-month follow-up under treatment.

## Ethics

The project is conducted in line with the principles of the Declaration of Helsinki, and was approved by the Ethics Committee of the University Hospital Center Zagreb and the Ethics Committee of the School of Medicine, University of Zagreb. Apart from the implemented genotyping, all other diagnostic and therapeutic procedures are in line with the standard of care for patients with respective diagnoses and treatments.

## Patients and patient management

Eligible individuals were adults (≥18 years of age) with indications for the use of CVDs of interest who provided informed consent for genotyping the genes of interest and for the use of anonymized data for research purposes and publishing in scientific journals. Exclusion criteria were ongoing or actively treated solid organ or hematological malignancies, advanced neurodegenerative disease, any form of dementia, severely advanced heart, kidney, or liver failure, having received a transplant, and life expectancy of less than one year.

The CVDs of interest were prescribed in line with their approved indications, whereas the individual drugs and posology were chosen at the discretion of the attending consultant. “Cases” enrolled at presentation with ADRs provided blood samples for genotyping and underwent standard clinical chemistry and biochemistry laboratory testing. Other diagnostic procedures, as well as decisions about discontinuation/switching of treatment, were performed at the discretion of the attending consultant. Detailed medical histories (in person, medical records) were taken and physical examination was performed.

Patients enrolled as “contemporary non-cases” underwent clinical (re)assessment, medical history taking/records review, and provided blood samples for genotyping and standard clinical chemistry/biochemistry laboratory tests. They were followed up through regular control visits (in 3-6-month intervals) and on an as-needed basis, and hospital information systems were periodically checked to record ADRs. If no ADRs occurred, the patients were re-assessed in person or by telephone contact at the end of the follow-up.

## Assessment of exposures

For the genetic analysis (PGx exposures), 3 mL of whole blood in an EDTA tube was collected, and genomic DNA was extracted using the QIAamp DNA Mini Kit (Qiagen, Hilden, Germany), according to the manufacturer's instructions. Pharmacogenetic analysis was performed using the TaqMan^®^ Real-Time PCR method on a PCR device in the 7500 Real-Time PCR System (Applied Biosystems, Foster City, CA, USA) using specific TaqMan^®^ DME Assay and TaqMan^®^ SNP Assay reagents (Applied Biosystems) and/or for genotyping polymorphisms of relevant pharmacogenes: CYP2C9 (*2, *3), CYP2C19 (*2, *3, *17), CYP2C18 (c.*31C>T), CYP2D6 (CNV, *3, *4, *5, *6, *9, *10, *41), CYP3A4 (*1B, *22), CYP3A5 (*3), CYP2J2 (*7, c.1331-2201T>C), CES1 (rs2244613, rs8192935), ABCB1 (c.1236C>T, c.2677G>T/A, c.3435C>T, rs4148738), ABCC2 (c.-24C>T, c.1249G>A), ABCG2 (c.34G>A, c.421C>A), SLCO1B1 (*c.388A>G, c.521C>T, c.463C>A, c.1929A>C*). Table 1 shows the genes of interest, SNP ID (rs), c. DNA, and *TaqMan^®^SNP* Assay ID.

**Table 1:** Pharmacogenes/variants of interest, single nucleotide polymorphism (SNP) identifiers (ID) (rs), c. DNA, and *TaqMan^®^SNP* Assay ID

Gene/variant	SNP ID	c. DNA	TaqMan®SNP Assay ID
CYP2C9*2	rs1799853	c.430C>T	C__25625805_10
CYP2C9*3	rs1057910	c.1075A>C	C__27104892_10
CYP2C19*2	rs4244285	c.681G>A	C__25986767_70
CYP2C19*17	rs12248560	c.-806 C > T	C____469857_10
*CYP2C:TG (CYP2C18)*	rs2860840	c.31C>T	C__11201742_10
*CYP2C:TG (CYP2C18)*	rs11188059	c.819+2182G>A	C__31983321_10
CYP2D6*3	rs35742686	c.775del	C__32407232_50
CYP2D6*4	rs3892097	c.506-1G>A	C__27102431_D0
CYP2D6*5	-	gene deletion	TaqMan® Copy Number Assay
CYP2D6*6	rs5030655	c.454del	C__32407243_20
CYP2D6*9	rs5030656	c.841_843del	C__32407229_60
CYP2D6*10	rs1065852	c.100C>T	C__11484460_40
CYP2D6*41	rs28371725	c.985+39G>A	C__34816116_20
*CYP2D6 *CNV exon 9	/	ex 9 CNV	Hs00010001_cn
*CYP2D6 *CNV intron 2	/	in 2 CNV	Hs04083572_cn
*CYP2D6 *CNV intron 6	/	in 6 CNV	Hs04502391_cn
CYP3A4*1B	rs2740574	c.-392A>G	C___1837671_50
CYP3A4*22	rs35599367	c.522-191C>T	C__59013445_10
CYP3A5*3	rs776746	c.219-237A>G	C__26201809_30
*CES1*	rs2244613	c.1168-33A>C	C__11290377_10
*CES1*	rs8192935	c.257+885T>C	C__26935203_10
*ABCB1*	rs1128503	c.1236C>T	C___7586662_10
*ABCB1*	rs2032582	c.2677G>T	C_11711720D_40
*ABCB1*	rs2032582	c.2677G>A	C_11711720C_30
*ABCB1*	rs1045642	c.3435C>T	C___7586657_20
*ABCB1*	rs4148738	c.2482-2236G>A	C___1253813_10
*ABCC2*	rs717620	c.-24C>T	C___2814642_10
*ABCC2*	rs2273697	c.1249G>A	C__22272980_20
*ABCG2 *	rs2231137	c.34G>A	ID AN49GAM
*ABCG2 *	rs2231142	c.421C>A	C__15854163_70
CYP2J2*7	rs890293	c.-76G>T	C___9581699_80
*CYP2J2*	rs11572325	/	C__30760106_10
*SLCO1B1 *	rs2306283	c.388A>G	C___1901697_20
*SLCO1B1 *	rs11045819	c.463C>A	ANERC7J PN
*SLCO1B1 *	rs4149056	c.521T>C	C__30633906_10
*SLCO1B1 *	rs34671512	c.1929A>C	C__25605954_10

Further genotyping was undertaken to detect potential predisposition to clotting disorders such as *FII (20210G>A, rs1799963), FV Leiden (1691G>A, rs6025),* and *MTHFR* (*677C>T, rs1801133, 1298A>C, rs1801131)*. DNA samples were also stored for eventual gene sequencing when considered relevant having in mind the complexity of ADRs and the inability to relate them to the standardly typed genes.

Pharmacological exposure refers to any other drugs used concomitantly with the CVDs of interest. Their possible perpetrator or victim roles were assessed using the Lexicomp^®^ Clinical Decision Support System. Lexicomp includes access to a database of monographs on medicines with the ability to simulate interactions between medicines, medicines and herbal preparations, and between herbal preparations, and categorizes them by clinical relevance (from “no clinical relevance” to “concomitant use contraindicated”) (59). In addition, the Flockhart Drug Interaction Table of Indiana University (IU) CYP450 (60) was used. Drug-drug-gene interactions were evaluated using open databases and literature that contain pharmacogenomic information to obtain verified translation of the genotype into a phenotype: PharmGKB, Flockhart Table (IU), SuperCyp, UpToDate, and SNPedia (61). Using a panel-consensus method, the likelihood of a relationship of an ADR with drug interactions was determined, and also whether this might depend on the pharmacogenes of interest (drug-drug-gene).

## Assessment of outcomes (adverse drug reactions)

Bleeding events associated with antiplatelet use were classified according to severity based on the European Society of Cardiology (ESC) (62,63) criteria as: a) trivial bleeding – does not require medical intervention and further evaluation; b) mild bleeding – requires medical attention but not hospitalization; c) moderate bleeding – causes hemoglobin to drop by more than 30 g/L and/or requires hospitalization, but is not hemodynamically threatening and does not develop rapidly; d) severe bleeding – requires hospitalization, causes hemoglobin to drop by more than 50 g/L, but is not hemodynamically threatening and does not develop rapidly; e) life-threatening bleeding – active bleeding that directly endangers the patient's life.

Bleeding events associated with DOACs are classified as: a) major bleeding (1. fatal bleeding and/or 2. symptomatic bleeding into a critical organ or region: intracranial, intraspinal, intraocular, retroperitoneal, intra-articular, pericardial, or intramuscular compartment syndrome, or 3. causes a hemoglobin drop of ≥20 g/L or requires 2 or more doses of erythrocyte concentrate or whole blood); b) clinically relevant non-major bleeding (1. spontaneous hematomas on the skin larger than 25 cm; 2. continuous nosebleeds longer than 5 minutes; 3. macroscopic hematuria, spontaneous or if associated with intervention, lasting more than 24 hours; 4. spontaneous rectal bleeding [more than spot bleeding]; 5. gingival bleeding for more than 5 minutes; 6. bleeding that causes hospitalization and/or surgery; 7. bleeding resulting in transfusion of fewer than 2 doses of erythrocyte concentrate or whole blood; 8. bleeding considered clinically relevant by the physician) (64-67).

Statin-related myotoxicity (SRM) is classified into seven phenotypic categories (11,68): a) SRM 0 represents asymptomatic elevations in serum creatine kinase (CK)<4 × the upper limit of reference interval (ULRI); b) SRM 1 and 2 are myalgia (pain, cramps, and/or weakness) without (SRM 1) or with mildly elevated CK (<4 × ULRI, SRM 2); c) SRM 3 represents myopathy with CK>4 × but <10 × ULRI; d) SRM 4 is severe myopathy with CK>10 × but <50 × ULRI; e) SRM 5 represents rare but potentially life-threatening rhabdomyolysis with CK>10 × ULRI, myalgia, and renal impairment or CK>50 × ULRI; f) SRM 6 consists of a very rare immune-mediated necrotizing myopathy, which persists despite discontinuation of statins (69,70).

The basis for the detection and classification of drug-induced liver injury (DILI) are serum aminotransferase values (ALT and AST), alkaline phosphatase, and total bilirubin. According to current recommendations, a minor increase in ALT or AST due to an adaptive and reversible response of the liver to statins or a consequence of an existing liver disease (ie, fatty liver) should not be classified as DILI (71). Since the signs of hepatic side effects of statins are usually mild, some authors define statin hepatotoxicity by liver function tests (AST and ALT) increasing above the upper limit of the reference interval (72). The liver enzymes are monitored in line with the 2019 ESC Guidelines on the treatment of dyslipidemia (73).

## Data analysis

The ADRs in focus of this project are known elements of the pharmacology of P2Y12 antiplatelets, DOACs, and statins – these drugs may cause the respective ADRs. However, in the target population defined as patients treated with these CVDs, the exposures of interest – PGx exposures (“pharmacogenes” presumably reflecting on the enzyme/transporter activity) and pharmacological exposures [pepetrator drugs]) – are also considered as causal factors: in people treated with the respective CVDs, these exposures may affect the likelihood of ADR occurrence by affecting, eg, CVD absorption, elimination (and, consequently, systemic exposure), and/or intracellular concentration, or by affecting the same biological functions but through different mechanisms. The assembled data provide a basis for a series of case-control type analyses, each focused on assessing a relationship of a particular exposure (eg, genotype, haplotype, diplotype, phenotype, one or more perpetrator drugs, and any combination thereof) with the occurrence of a particular ADR (relevant for the respective CVDs). Considering the mode of data generation and the nature of the exposures of interest (ie, present before the occurrence of the outcome), potentially observed outcome-exposure associations are likely to be causal, provided that confounding is reasonably controlled. Various weighting and matching methods are used to achieve balance between cases and controls on potential confounders when assessing a relationship between a particular exposure and the outcome (ie, other PGx and/or pharmacological exposures, comorbidities, demographics, renal function).

## Biobank repository

Among other objectives, the project was initiated with the intention to start a Biobank, ie, a repository of DNA samples and a database of relevant clinical data, including the characterization of ADRs.

## Expected outcomes and implications

Although PGx research of CVDs has yielded significant results with translation into clinical use, this is not the case for newer therapies. The existing pharmacogenetic recommendations are based mainly on the evaluation of individual drug-gene interactions. Therefore, it is necessary to develop procedures and methods that better reflect the real-life situation of patients with comorbidities and polytherapy (particularly the elderly), and might better predict interactions of multiple drugs and genes that could affect the frequency and severity of the ADRs. The ethnic and racial disparities in the prevalence of particular “pharmacogenes” are well known, and although European populations share many common characteristics, the discrepancy between them might still exist. The PGx-CardioDrug project is, to our knowledge, the first of this kind focused on a European population of Slavic descent.
